# 

**Published:** 1788

**Authors:** 


### C O N T E N T S.

Page

I. AN Account of three Cases, viz. 1. of the Extraction and Depression of the Cataract in the same Patient; 2. of an encysted Hydrocele of the Tunica communis of the spermatic Chord, communicating with the Tunica Vaginalis Testis; and 3. of an Amputation below the Knee, the Event of which shows that the Advantages of the Union by the first Intention, after such an Operation, are as easily obtained in the Leg as in the Thigh. By Mr. Richard Sparrow, one of the Surgeons of the Charitable Infirmary at Dublin. - - - - 109

II. An Account of a painful Swelling of the Perinæum, which took place immediately after delivery, and terminated in Sphacelus. By Mr. Thomas Reeve, Surgeon at Boterdale in Suffolk. - - - - - - 119

III. Hints respecting the Hydrocephalus internus. By John Warren, M. D. Physician at Taunton, honorary Member of the Medical Society at Edinburgh, corresponding Member of the Royal Medical Society at Paris, and an Associate of the Academies at Bologna, Florence, and Rome. - - - - 122

Page

IV. *Cases of the spontaneous Cure of Aneurism, with Remarks. By Mr.* Edward Ford, *Surgeon to the Westminster General Dispensary.* 142

V. *Observations on sudden Deaths occasioned by a Rupture of the left Ventricle of the Heart. By M.* Portal, *Professor of Anatomy, and Member of the Royal Academy of Sciences at Paris, &c. Translated from the French.* 156

VI. *Observations on some Causes of the Excess of the Mortality of Males above that of Females. By* Joseph Clarke, *M. D. Physician to the Lying-in Hospital at Dublin. Communicated by the Rev.* Richard Price, *D. D. F.R.S. in a Letter to* Charles Blagden, *M. D. Sec. R. S.—From the Philosophical Transactions of the Royal Society of London.* 179

VII. *Experiments made to determine the positive and relative Quantities of Moisture absorbed from the Atmosphere by various Substances, under similar Circumstances. By Sir* Benjamin Thompson, *Knt. F. R. S.—From the Philosophical Transactions of the Royal Society of London.* - - - - - 201

Catalogue of Books. - - 206

